# Predictors of Effectiveness of Platelet-Rich Plasma Therapy for Knee Osteoarthritis: A Retrospective Cohort Study

**DOI:** 10.3390/jcm10194514

**Published:** 2021-09-29

**Authors:** Yoshitomo Saita, Yohei Kobayashi, Hirofumi Nishio, Takanori Wakayama, Shin Fukusato, Sayuri Uchino, Yasumasa Momoi, Hiroshi Ikeda, Kazuo Kaneko

**Affiliations:** 1 Department of Sports and Regenerative Medicine, Juntendo University, 2-1-1 Hongo, Bunkyo-ku, Tokyo 113-0033, Japan; yhkobaya@juntendo.ac.jp (Y.K.); jmp4ubitj@gmail.com (H.N.); twakaya@juntendo.ac.jp (T.W.); s-fukusato@juntendo.ac.jp (S.F.); s-uchino@juntendo.ac.jp (S.U.); y-momoi@juntendo.ac.jp (Y.M.); 2 Department of Orthopaedics and Sports Medicine, Juntendo University, 2-1-1 Hongo, Bunkyo-ku, Tokyo 113-0033, Japan; hi-ikeda@juntendo.ac.jp (H.I.); k-kaneko@juntendo.ac.jp (K.K.)

**Keywords:** knee osteoarthritis, platelet-rich plasma, predictor of effectiveness

## Abstract

There has recently been growing interest worldwide in biological therapies such as platelet-rich plasma injection for the treatment of knee osteoarthritis. However, predicting the effectiveness of platelet-rich plasma therapy remains uncertain. Therefore, this retrospective cohort study was performed to assess a range of predictors for the effectiveness of platelet-rich plasma therapy in treating knee osteoarthritis. The study included 517 consecutive patients who underwent three injections of leucocyte-poor platelet-rich plasma therapy from 2016 to 2019 at a single institution. The treatment outcomes, including patient-oriented outcomes (visual analogue scale score and Knee Injury and Osteoarthritis Outcome Score), were analyzed and compared according to the severity of knee osteoarthritis based on Kellgren–Lawrence (KL) grading using standing plain radiographs. Fisher’s exact test, univariate regression, and multivariate regression were used for data analysis. Patient-oriented outcomes were significantly improved 6 and 12 months after platelet-rich plasma therapy. The overall responder rate in patients who met the Outcome Measures in Rheumatology (OMERACT)–Osteoarthritis Research Society International (OARSI) responder criteria was 62.1%. The responder rate was significantly lower in patients with severe knee osteoarthritis (KL4, 50.9%) than in those with mild (KL2, 75.2%) and moderate (KL3, 66.5%) knee osteoarthritis. The multivariate logistic regression analysis revealed that deterioration of the knee osteoarthritis grade (increased KL grade) was a significant predictor of a worse clinical outcome (odds ratio, 0.58; 95% confidence interval, 0.45–0.75; *p* < 0.001). The relative risk for non-responders in severe (KL4) KOA was 2.1 (95% CI, 1.5–3.0) at 6 months and 2.3 (1.6–3.2) at 12 months compared with mild-to-moderate (KL2-3) KOA. The efficacy of platelet-rich plasma therapy was not affected by age, sex, body weight, or platelet count. This study revealed that the effectiveness of platelet-rich plasma therapy for the treatment of knee osteoarthritis is approximately 60% and that the effectiveness depends on the severity of knee osteoarthritis. This observation is useful not only for physicians but also for patients with knee osteoarthritis.

## 1. Introduction

Osteoarthritis is a major public health concern worldwide because it is associated with considerable disability. Conservative management options for knee osteoarthritis (KOA) include rehabilitative and orthotic therapies and treatment with analgesics and anti-inflammatory agents [1]. Orthopedic surgeons usually recommend conservative treatments, such as rehabilitative therapies, non-steroidal anti-inflammatory drugs, steroid injections, and hyaluronic acid (HA) injections before offering surgery [2]. If these conservative therapies fail, surgical treatments such as osteotomy around the knee joint and arthroplasty should be considered.

A new category of conservative and regenerative treatments involving cell-based therapies, such as platelet-rich plasma (PRP) therapy, has recently been introduced [3]. PRP therapy is the most frequently used cell-based therapy for KOA because it is simple, minimally invasive, inexpensive, and has fewer complications than arthroplasty [3]. A meta-analysis of clinical trials involving intra-articular (IA) PRP injection in patients with KOA demonstrated that PRP has favorable effects on improving pain and functional scores compared with HA, steroid, and saline injections [4,5]. A meta-analysis of 21 randomized controlled clinical trials (RCTs) demonstrated that a clinically important benefit for pain relief was seen for intra-articular PRP compared with intra-articular saline (standardized mean difference (SMD) = −1.38 (95% CI, −2.07 to −0.70); *p* < 0.0001; I 2 = 37%) and corticosteroid solution injections (SMD = −2.47 (95% CI, −3.34 to −1.61); *p* < 0.00001; I 2 = 47%) [4]. In a previous RCT investigating the efficacy of PRP, the responder rate of Outcome Measures in Rheumatology (OMERACT)-Osteoarthritis Research Society International (OARSI) responder criteria was 65.5% at 12 months [6]. Conversely, the OARSI guidelines on developing patient-focused treatment recommendations do not recommend PRP therapy because the evidence in support of this treatment is of low quality [7]. Most studies to date treated only patients with mild to moderate KOA and compared the effectiveness of PRP therapy with other treatments such as HA injection [4,8]. In the clinical setting, however, many patients with severe KOA wish to undergo PRP therapy as well. In addition, factors that predict the effectiveness of PRP therapy for KOA remain unknown. Therefore, we performed the present retrospective study to confirm whether the clinical outcomes of PRP therapy for KOA are affected by the patients’ characteristics, including age, sex, body mass index, severity of KOA, and platelet concentration.

## 2. Materials and Methods

### 2.1. Patients

The clinical records of 517 consecutive patients who underwent PRP therapy for the treatment of chronic symptomatic KOA from 2017 to 2019 at our hospital were reviewed. The outcome measuring was conducted prospectively based on the protocol approved by a law regulating the safety of regenerative medicine in Japan (approval number PB3150023). The electronic clinical records of these patients were reviewed retrospectively. The study was approved by the ethics committee of our hospital. The indication for PRP therapy at our hospital is chronic knee pain for at least 1 year despite other known conservative treatments, such as rehabilitation, oral non-steroidal anti-inflammatory drugs, IA injection of HA, or corticosteroids. Written informed consent was obtained from all patients before the initiation of PRP therapy. The exclusion criteria for PRP therapy were systemic inflammatory diseases, such as rheumatoid arthritis and active infectious diseases; immunosuppression, a history of cancer, or poorly controlled diabetes mellitus; and platelet disorders or diseases. The severity of KOA was not an exclusion criterion. Information about each patient’s clinical course was evaluated, including data regarding patient-oriented outcomes, such as the visual analogue scale (VAS) score and Knee Injury and Osteoarthritis Outcome Score (KOOS), at the initiation of treatment and at 6 and 12 months after treatment. If both knees were affected, VAS in both knees were evaluated and the data collected were applicable to the more symptomatic side. The effectiveness of PRP therapy and various predictors of a good clinical outcome were analyzed in all 517 patients after the initiation of PRP therapy. The analysis to explore the predictors of the effectiveness of PRP therapy was performed using 6- and 12-month timepoint data.

### 2.2. Outcomes

Clinical outcomes were assessed based on the transition of the VAS score and KOOS. The KOOS is a self-administered patient-reported outcome measure with individual items graded on a 5-point Likert scale from 0 to 4, which comprises the following five subscales: pain, symptoms, activities of daily living, sport and recreation function, and knee-related quality of life [9]. The effectiveness of PRP therapy was determined using the OMERACT-OARSI responder criteria [10], which are based on a combination of absolute and relative changes in pain, function, and patient global assessment. The patients were classified as responders if one of the following two criteria was fulfilled: (1) high improvement in pain: ≥50% improvement + absolute change of ≥20 in pain; or (2) improvement in at least two of the following: ≥20% improvement + absolute change of ≥10 in pain, ≥20% improvement + absolute change of ≥10 in function, or ≥20% improvement + absolute change of ≥10 in the patient global assessment of disease activity.

### 2.3. PRP Preparation

The protocol and ethics of PRP therapy were certified by a special committee for regenerative medicine based on a law regulating the safety of regenerative medicine in Japan (approval number PB3150023). The PRP preparation was obtained by a single centrifugation of whole blood using the MyCells autologous platelet preparation system (Kaylight Ltd., Ramat HaSharon, Israel). A total of 22 mL of whole blood was aspirated from the median cubital vein, and 4.0 to 5.0 mL of PRP was obtained according to the manufacturer’s instructions. In brief, 22 mL of whole blood was aspirated into the two sets of MyCells kit syringes containing 1 mL of anticoagulant dextrose solution A and separation gel. Next, the samples were centrifuged for 7 min at 2000× *g*. After aspirating the supernatant platelet-poor plasma, the residual 2.0 to 2.5 mL of plasma was pipetted to peel off the platelets from the surface of the separation gel. The filter column was then inserted into the separation syringe to remove the debris and filtered PRP. The PRP obtained using this method is classified as P2-Bβ PRP (leucocyte-poor (LP)-PRP) based on the PAW classification system [11]. The total cost of one PRP injection at our hospital was 25,000 yen during this study period.

### 2.4. PRP Injection

The patient was placed in the supine position, and the knee was extended or slightly bent, depending on the restriction of his or her range of motion. The skin was washed with a povidone-iodine solution. PRP was injected into the suprapatellar bursa via the lateral suprapatellar approach using a 21-gauge needle. The superior lateral aspect of the patella was palpated and the needle inserted 1 cm above and 1 cm laterally. The needle was tilted beneath the patella at a 45-degree angle. If the joint fluid could be aspirated, it was removed before injecting the PRP. According to our standard protocol, 4 to 5 mL of LP-PRP was injected three times every 4 weeks. Adverse effects, such as infection after PRP therapy, were evaluated after PRP therapy.

### 2.5. Radiological Analyses

A standing anteroposterior radiograph was obtained to evaluate the grade of KOA. KOA was assessed using the Kellgren–Lawrence (KL) classification system [12] as follows: 0 = normal; 1 = doubtful joint space narrowing and possible osteophytes; 2 = definite osteophytes and possible joint space narrowing; 3 = moderate multiple osteophytes, definite joint space narrowing, some sclerosis, and possible deformity of bone ends; and 4 = large osteophytes, marked joint space narrowing, severe sclerosis, and definite deformity. The anatomic femorotibial angle was calculated using anatomical axes of the femur and tibia on the standing anteroposterior radiograph.

### 2.6. Statistical Analyses

Fisher’s exact test was performed to compare each KL grade and thus confirm whether the efficacy of PRP therapy was dependent upon the severity of KOA. Univariate regression was performed to determine whether the independent variables (age, sex, body mass index, severity [grade] of KOA, and platelet count) were related to the dependent variable (PRP therapy responder who meets the OMERACT-OARSI responder criteria). Multivariate regression was then performed based on the results of univariate regression and clinical importance, considering multicollinearity. In the multivariate logistic regression analysis, the FTA was not included as a dependent variable because it is associated with the KL grade. All statistical analyses were performed using SPSS version 20.0 (IBM Corp., Armonk, NY, USA). All *p*-values were two-sided, and *p*-values of <0.05 were considered statistically significant.

## 3. Results

### 3.1. Patient Characteristics

Table 1 shows the patients’ demographics according to the severity of KOA. Patients with KL grade 2 KOA were significantly younger than those with KL grades 3 and 4 KOA (*p* < 0.001), and patients with KL grade 3 KOA were significantly younger than those with KL grade 4 KOA (*p* = 0.002). The female sex was dominant in all KL grades, but the ratio of male patients was significantly higher than that of female patients in the group with lower grades of KOA. The femorotibial angle (FTA) significantly increased as the KOA grade became more severe. The platelet count did not differ across all KL grades in both peripheral blood and PRP.

### 3.2. Observed Outcomes after PRP According to Patient-Oriented Outcomes and Severity of KOA

**Figure 1:**
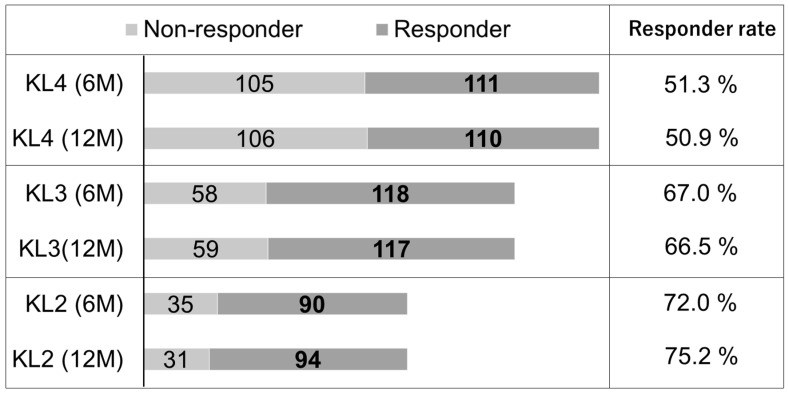
OMERACT-OARSI responder criteria-based responder rate at 12 months.

**Figure 2:**
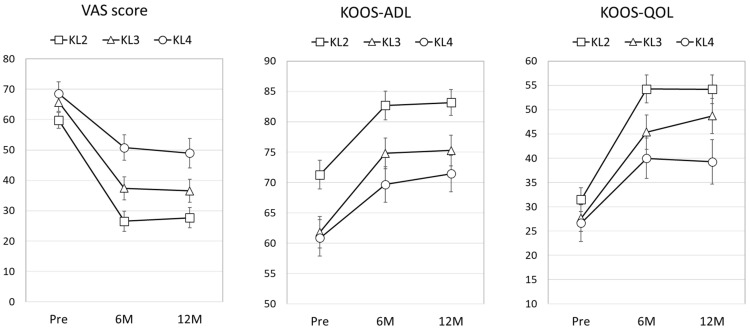
Transition of patient-oriented outcomes. Data represent the average. Error bars represent 95% confidence intervals. VAS, visual analogue scale; KOOS, Knee Injury and Osteoarthritis Outcome Score; ADL, activities of daily living; QOL, quality of life; KL, Kellgren–Lawrence; Pre, pretreatment; M, months.

The clinical course from treatment initiation to 12 months after PRP therapy was analyzed to determine the efficacy of PRP therapy. The overall responder rate of those who met the OMERACT-OARSI responder criteria at 12 months was 62.1% (318 responders among 517 patients). The responder rates among patients with mild (KL grade 2), moderate (KL grade 3), and severe (KL grade 4) KOA were 75.2% (95% confidence interval (CI), 67.5–82.9), 66.5% (59.4–73.5), and 50.9% (44.2–57.6) respectively (Figure 1). The responder rate was significantly higher in patients with mild to moderate KOA than in those with severe KOA, and it was highest in patients with mild KOA. The transitions of the VAS score, KOOS activities of daily living subscale, and KOOS quality of life subscale are shown in Figure 2. Patient-oriented outcomes were significantly improved at 6 and 12 months after the PRP injections in all groups (*p* < 0.001). The multivariate logistic regression analysis revealed that deterioration of the KOA grade was a significant predictor of a worse clinical outcome of PRP therapy (odds ratio (OR), 0.58; 95% CI, 0.45–0.75; *p* < 0.001) (Table 2). The relative risk for non-responders in severe (KL4) KOA was 2.1 (95%CI, 1.5–3.0) at 6 months and 2.3 (1.6–3.2) at 12 months compared with mild-to-moderate (KL2-3) KOA. Regarding other predictor variables, higher body weight tended to predict a poorer response to PRP therapy (OR, 0.98; 95% CI, 0.97–1.00; *p* = 0.10), but it was not statistically significant. Interestingly, the platelet count both in peripheral blood and PRP was not associated with the effectiveness of PRP therapy. Neither soft tissue infection around the injection site nor knee joint infection was observed during this period.

The responder rate was highest in mild (KL grade 2) knee osteoarthritis. OMERACT-OARSI, Outcome Measures in Rheumatology–Osteoarthritis Research Society International; KL, Kellgren–Lawrence; 6M, 6 months; 12M, 12 months.

### 3.3. Influence of Malalignment on Effectiveness of PRP Therapy in Severe (KL Grade 4) KOA

We considered the possibility that malalignment would influence the effectiveness of PRP therapy. Therefore, the logistic regression was performed in each KL grade. In KL grades 2 and 3 KOA, the FTA was not associated with the effectiveness (responder) of PRP therapy (OR, 1.02; 95% CI, 0.89–1.16 and OR, 0.96; 95% CI, 0.89–1.03, respectively). However, in KL grade 4 KOA, the FTA was significantly associated with the effectiveness of PRP therapy (OR, 0.95; 95% CI, 0.91–0.98; *p* = 0.01). Even in patients with severe KOA (KL grade 4), PRP showed good effectiveness (responder rate of 60.0%–85.7%) in patients with an FTA of <180 degrees, while it showed poor effectiveness (responder rate of 28.1%) in those with an FTA of >190 degrees (Table 3).

## 4. Discussion

This study demonstrated that the progression of the severity of radiographic KOA was a significant predictor of a poor outcome for a course of PRP therapy. This seems natural because if the KOA worsens, the efficacy of PRP therapy may decrease. Interestingly, most previous studies concluded that the severity of KOA was not associated with the efficacy of PRP therapy [4,5,13]. This could be attributed to the fact that most previous prospective studies excluded patients with severe KOA (KL grade 4). In contrast, the present retrospective cohort study included not only patients with mild to moderate KOA (KL grades 2 and 3) but also those with severe KOA (KL grade 4). The inclusion of patients with KL grade 4 KOA is important because it showed that PRP therapy is more effective in the early stages of KOA, suggesting that early intervention could reduce the need for future surgical treatments such as knee joint replacement. This point is clinically noteworthy because most patients desire to attempt PRP therapy when traditionally recommended treatments have failed. However, our results suggest that mild to moderate KOA is a good clinical indication for PRP therapy. This result is informative not only for physicians but also for patients with KOA as they consider the indication and timing of initiating PRP therapy.

The mechanisms through which PRP injections improve the symptoms of KOA remain unclear. However, previous experimental studies have suggested that PRP promotes extracellular matrix formation in human articular chondrocytes [14] and inhibits inflammatory processes in osteoarthritic chondrocytes [15]. Thus, IA injection of PRP may possess both chondroregenerative and anti-inflammatory effects [16]. Several reports have investigated changes in articular cartilage conditions after PRP injection, but most studies concluded that articular cartilage did not improve after PRP [17,18]. A recent double-blind randomized controlled trial conducted by Raeissadat et al. [19] revealed that the patellofemoral cartilage volume and synovitis significantly improved in PRP-treated patients with KOA based on magnetic resonance imaging analyses performed 8 months after treatment. This finding suggests that PRP can regenerate the articular cartilage of the knee joint, especially in mechanically stable conditions. Indeed, the effectiveness of PRP therapy was low in patients with severe (KL grade 4) KOA compared with mild to moderate (KL grades 2 and 3) KOA in this study (Figure 1); even in severe KOA, however, the effectiveness was not low in patients with an FTA of <180 degrees (Table 3). Therefore, in mechanically unstable conditions, a combination of PRP therapy plus surgical treatments to stabilize the knee joint, such as meniscal repair, ligament reconstruction, and osteotomy around the knee joint, would synergistically improve the efficacy of PRP therapy.

Kobayashi et al. [20] reported that leucocyte-rich PRP (LR-PRP) contains high levels of matrix metalloprotease 9, which causes degradation of the articular cartilage, compared with LP-PRP. In this study, we used LP-PRP because it was hypothesized to be more suitable for IA injection than LR-PRP in the treatment of KOA. However, Riboh et al. [21] analyzed randomized controlled trials (evidence level 1) and three prospective comparative studies (evidence level 2) and concluded that the efficacy and adverse effects of PRP therapy were similar between LP- and LR-PRP. In addition, Kenmochi [22] reported that LR-PRP therapy for KOA in Japanese patients was safe and effective. Therefore, the effectiveness of PRP therapy for the treatment of KOA could be considered similar at least when using these two types of PRPs at this time. Notably, the present study revealed no relationship between the concentration of platelets and effectiveness of PRP therapy (Table 2). Boswell et al. [23] reported that increasing the platelet concentration in PRP to decrease collagen gene synthesis and thus reduce leucocytes to minimize catabolic signaling would be more important than increasing platelets in an effort to maximize anabolic signaling. These findings suggest that the effectiveness of PRP is related to the quality of PRP, such as the concentration and variation of growth factors, rather than to the concentration of platelets.

From a health economics perspective, the cost of a single LP-PRP injection in this study was 25,000 yen (approximately 200 US dollars); thus, the summation of three injections was 75,000 yen (approximately 600 US dollars) at our institute. The cost for PRP therapy depends on the use of a PRP-preparing system, the cost of which varies from 7000 to 200,000 yen (approximately 56 to 1600 US dollars). Therefore, the cost for PRP therapy varies among clinics and hospitals in Japan from approximately 20,000 to 350,000 yen (approximately 160 to 2800 US dollars) and is influenced by the type of PRP-preparing kit that is used; notably, these costs are not covered by the national health insurance system in Japan. Although the health insurance system is different in every country, it is worthwhile for clinicians to consider PRP therapy as a conservative treatment option before offering surgical treatments, particularly to patients with mild to moderate deformity, patients who are too young to undergo joint replacement, and patients with high-risk comorbid conditions, such as cardiovascular disease.

The main limitations of this study are its retrospective design and lack of a control treatment group. Although this study included patients with chronic knee pain who did not respond to other known conservative treatments for more than 1 year, the effectiveness of the PRP therapy itself could have partially been a result of the placebo effect. However, a retrospective study is acceptable for assessing the predictors of the effectiveness of PRP therapy and the extent to which its effectiveness depends on the severity of KOA.

## 5. Conclusions

In conclusion, this study revealed that the effectiveness of PRP therapy for the treatment of KOA was approximately 60% and that severe knee deformity was a significant predictor of lesser improvement in patient-oriented outcomes. Age, sex, and the platelet concentration were not associated with the effectiveness of PRP therapy. Severe varus deformity with an FTA of >190 degrees in patients with KL grade 4 KOA diminished the effectiveness of PRP therapy.

## Figures and Tables

**Table 1:** Patient demographics.

KL Classification	Total	KL Grade 2	KL Grade 3	KL Grade 4
Number of knees	517	125	176	216
Age (years)	70.0 (69.1–70.9)	65.4 (63.4–67.5)	70.3 (69.1–71.5)	73.2 (71.9–74.4)
Sex, male/female (number of patients)	135/382	43/72	42/134	43/173
Height (cm)	157.6 (157.6–159.2)	162.6 (160.9–164.3)	158.5 (157.2–159.8)	155.8 (154.7–156.9)
Body weight (kg)	62.4 (60.5–62.7)	63.6 (60.9–66.2)	61.5 (59.7–63.3)	60.5 (58.9–62.1)
Body mass index (kg/m^2^)	25.0 (24.1–24.8)	23.8 (23.1–24.5)	24.4 (23.8–24.9)	24.9 (24.3–25.4)
Femorotibial angle (degrees)	180.6 (180.0–181.0)	177.5 (176.8–178.1)	179.4 (178.7–180.0)	183.2 (182.3–184.1)
Platelet count (whole blood, ×10^9^/L)	221.4 (213.9–224.7)	219.3 (208.8–229.7)	221.1 (212.1–230.1)	218.1 (209.0–227.1)
Platelet count (PRP, ×10^9^/L)	475.4 (447.7–478.3)	461.8 (429.4–494.2)	463.2 (437.1–489.3)	459.0 (434.7–483.3)

Data are presented as average (95% confidence interval) unless otherwise indicated. KL, Kellgren–Lawrence; PRP, platelet-rich plasma.

**Table 2:** Logistic regression analysis to identify predictors of a good clinical outcome (responders).

Predictors	Univariate		Multivariate	
	OR (95% CI)	*p*-Value	OR (95% CI)	*p*-Value
Age	0.99 (0.97–1.01)	0.12	0.99 (0.97–1.01)	0.53
Sex	1.08 (0.72–1.62)	0.71	1.09 (0.68–1.78)	0.71
Body weight	0.98 (0.97–1.00)	0.15	0.98 (0.97–1.00)	0.10
Kellgren–Lawrence classification	0.58 (0.45–0.73)	<0.001	0.58 (0.45–0.75)	<0.001
Platelet count	1.00 (0.99–1.00)	0.57	1.00 (0.99–1.00)	0.74

OR, odds ratio; CI, confidence interval. Bold *p*-values are statistically significant.

**Table 3:** Femorotibial angle and responder rate in severe (KL grade 4) knee osteoarthritis.

Femorotibial Angle, Degrees	Responder Rate (Responders/Number of Patients)
<175	12/20 (60.0%, 36.5–83.5)
175–179	18/21 (85.7%, 69.4–102.0)
180–184	39/78 (50.0%, 38.7–61.3)
185–189	32/65 (49.2%, 36.7–61.7)
≥190	9/32 (28.1%, 11.7–44.6)

KL, Kellgren–Lawrence. Data represent (%, 95%CI).

## Data Availability

The data presented in this study are available on request from the corresponding author. The data are not publicly available due to protection of patients’ privacy.
